# The TIE1 transcriptional repressor controls shoot branching by directly repressing BRANCHED1 in Arabidopsis

**DOI:** 10.1371/journal.pgen.1007296

**Published:** 2018-03-23

**Authors:** Yan Yang, Michael Nicolas, Jinzhe Zhang, Hao Yu, Dongshu Guo, Rongrong Yuan, Tiantian Zhang, Jianzhao Yang, Pilar Cubas, Genji Qin

**Affiliations:** 1 State Key Laboratory of Protein and Plant Gene Research, School of Life Sciences, School of Advanced Agricultural Sciences, Peking University, Beijing, The People’s Republic of China; 2 Ocean-X Institute, SUSTech Academy for Advanced Interdisciplinary Studies, Southern University of Science and Technology, Shenzhen, Guangdong, The People’s Republic of China; 3 Plant Molecular Genetics Department, Centro Nacional de Biotecnología/CSIC, Campus Universidad Autόnoma de Madrid, Madrid, Spain; Stanford University School of Medicine, UNITED STATES

## Abstract

Shoot branching is a major determinant of plant architecture and is regulated by both endogenous and environmental factors. *BRANCHED1* (*BRC1*) is a central local regulator that integrates signals controlling shoot branching. So far, the regulation of BRC1 activity at the protein level is still largely unknown. In this study, we demonstrated that TIE1 (**T**CP **i**nteractor containing **E**AR motif protein **1**), a repressor previously identified as an important factor in the control of leaf development, also regulates shoot branching by repressing BRC1 activity. *TIE1* is predominantly expressed in young axillary buds. The gain-of-function mutant *tie1-D* produced more branches and the overexpression of *TIE1* recapitulated the increased branching of *tie1-D*, while disruption of *TIE1* resulted in lower bud activity and fewer branches. We also demonstrated that the TIE1 protein interacts with BRC1 *in vitro* and *in vivo*. Expression of BRC1 fused with the C-terminus of the TIE1 protein in wild type caused excessive branching similar to that observed in *tie1-D* and *brc1* loss-of-function mutants. Transcriptome analyses revealed that *TIE1* regulated about 30% of the *BRC1*-dependent genes, including the BRC1 direct targets *HB21*, *HB40* and *HB53*. These results indicate that TIE1 acts as a positive regulator of shoot branching by directly repressing BRC1 activity. Thus, our results reveal that *TIE1* is an important shoot branching regulator, and provide new insights in the post-transcriptional regulation of the TCP transcription factor BRC1.

## Author summary

Shoot branching is a key factor that not only affects plant survival but also determines food productivity in crop species. *BRANCHED1* (*BRC1*) integrates internal and external signals to determine shoot branching. However, the regulation of BRC1 at the protein level remains elusive. We found that TIE1 (**T**CP **i**nteractor containing **E**AR motif protein **1**) plays an important role in the control of shoot branching in Arabidopsis. Higher *TIE1* expression levels lead to bushier Arabidopsis plants. TIE1 directly interacts with BRC1 and represses BRC1 transcriptional activity. Furthermore, BRC1 downstream target genes are downregulated by TIE1. Our findings demonstrate that TIE1 acts as a key repressor of BRC1 activity and positively regulates shoot branching.

## Introduction

Shoot branching greatly affects plant architecture, one of the most important agronomic traits. The manipulation of shoot branching patterns is an efficient way to promote and manage crop production [1]. Shoot branching is a developmental process with a high plasticity and tightly regulated by diverse endogenous and environmental stimuli. The development of shoot branches starts from the initiation of axillary meristems (AMs) in the leaf axils. The AMs then develop into small buds with a few leaves, which either remain dormant or grow to form branches in response to internal or external cues [2].

Genetic analyses have identified several important transcriptional regulators, which form a complex regulatory network during the initiation of AMs [3]. However, few transcriptional regulators have been found to control local bud activity. *TEOSINTE BRANCHED1* (*TB1*) is an important domestication gene of maize that plays a central role in the control of shoot branching. *TB1* is a founder member of the TCP (TB1/CYCLOIDEA/PCF) family of transcription factors conserved in the plant kingdom. In both monocots and dicots, orthologs of *TB1* play a pivotal role in the control of bud activity. Examples of this are the rice *FINE CULM 1*/*OsTB1*, sorghum *SbTB1*, Arabidopsis *BRANCHED1 (BRC1)*, and tomato, pea and potato *BRC1-*like genes [4–9]. The Arabidopsis *BRC1* gene is predominantly expressed in developing axillary buds (axillary meristems, bud leaf primordia and subtending vascular tissue) and its expression levels decrease as buds grow out. *BRC1* acts as a suppressor of bud activity: loss-of-function *brc1* mutants display accelerated initiation of axillary meristem formation, faster bud development and more branches [6,10].

Increasing evidence indicates that *BRC1* is an integrator of diverse internal and external signals that control bud activity. The branch-suppressing hormone strigolactone (SL) controls shoot branching in part by positively regulating *BRC1* at the transcriptional level in Arabidopsis and pea [6,8,10–12]. The *brc1* mutants are insensitive to SL treatments and epistatic to *smxl6 smxl7 smxl8* triple mutants [8,13, 14]. On the contrary, the hormone cytokinin (CK) negatively regulates *BRC1* expression and promotes shoot branching in rice and pea [8,15], although the branching of *Psbrc1* pea mutants still respond to CK treatments [8]. Likewise, sugar is an important nutritional and signaling element proposed to be necessary for axillary bud outgrowth. *BRC1* transcript levels are reduced after sucrose application to buds [16–18], whereas low sucrose levels upregulate *TB1* expression in wheat [19]. In addition to the endogenous signals, *BRC1* is also regulated by numerous external inputs. For example, changes in light quality (i.e. a reduction in the red-to-far red light ratio) upregulate *BRC1* and lead to suppression of bud growth [20,21].

A *TB1* upstream regulator, IDEAL PLANT ARCHITECTURE1 (IPA1), has been identified in rice. IPA1 is a transcription factor that promotes the expression of *OsTB1* by directly binding to its promoter region [22]. *TB1* downstream targets [23,24] and also BRC1 targets begin to be characterized. Three HD-ZIP transcription factor-encoding genes, *HOMEOBOX PROTEIN* (*HB*) *21*, *HB40* and *HB53* have been shown to be directly regulated by BRC1 in Arabidopsis [18]. These HD-ZIPs and BRC1 together, upregulate *9-CIS-EPOXICAROTENOID DIOXIGENASE 3* (*NCED3*), which encodes a key enzyme in ABA biosynthesis to promote bud dormancy. However, although the transcriptional regulation upstream and downstream of *BRC1* begins to be understood, the regulation of BRC1 activity at the protein level is still very poorly known.

TCP Interactor containing EAR motif protein 1 (TIE1) was identified as a nuclear transcriptional repressor that regulates leaf development [25]. Overexpression of *TIE1* in the activation-tagging mutant *tie1-D* causes hyponastic leaves, while the disruption of *TIE1* leads to epinastic leaves. The TIE1 protein interacts with CIN-like TCP transcription factors and it also recruits the transcriptional corepressors TOPLESS (TPL)/TOPLESS-RELATED (TPR). Formation of this protein complex leads to a repression of the activity of CIN-like TCP transcription factors. The association of TIE1 with these TCPs further leads to an altered expression of TCP target genes, such as *LOX2*, *AS1* and *IAA3*. However, *tie1-D* and *jaw-D*, in both of which the TCP activity was downregulated, did not display completely identical phenotypes, which indicates that TIE1 may also bind other transcription factors and regulate additional biological processes. In addition, TIE1 is regulated by E3-ligase proteins termed TIE1-associated RING-type E3 ligases (TEARs) [26]. TEARs interact with TIE1 and are responsible for TIE1 degradation, which boosts CIN-like TCP activity during leaf development.

Here, we report that the transcriptional repressor TIE1 positively controls shoot branching by directly regulating BRC1 protein activity. We demonstrate that overexpression of *TIE1* leads to higher bud activity and more branches, whereas disruption of *TIE1* causes reduced bud activity and branch suppression. *TIE1* is predominantly expressed in axillary buds and is negatively regulated as buds grow out. TIE1 represses the expression of BRC1 target genes, probably by directly interacting with BRC1 and antagonizing its activity. Our data reveals a novel molecular mechanism by which plants control BRC1 activity accurately and flexibly via TIE1 at the protein level to determine bud activity in response to endogenous and environmental cues.

## Results

### Overexpression of *TIE1* causes excessive branching

**Fig 1:**
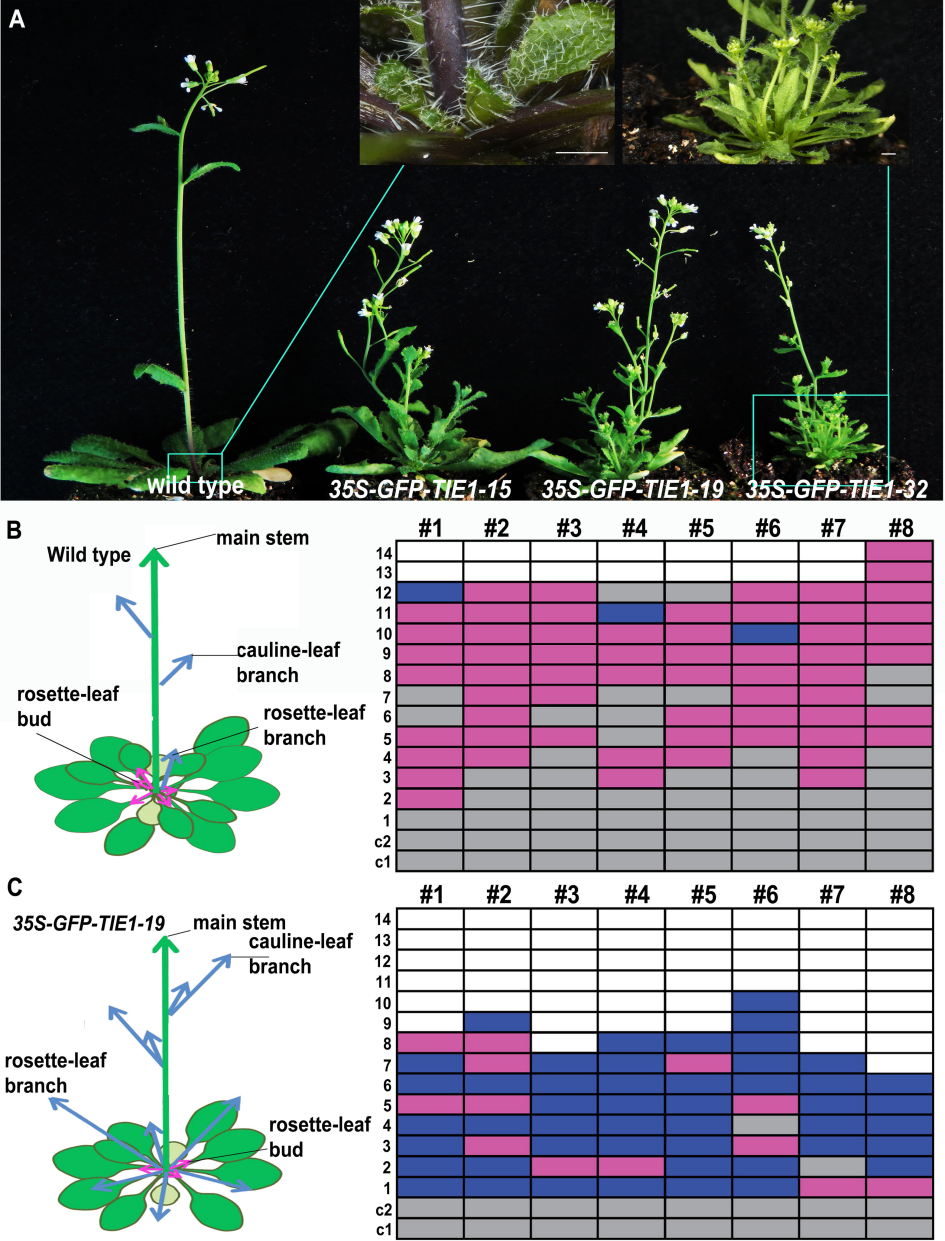
Overexpression of *TIE1* leads to excessive branching. (A) Branching phenotypes of 40-day-old wild-type plants and representative individuals of three independent *35S-GFP-TIE1* lines. The *35S-GFP-TIE1* mutants produce more branches than wild-type plants. Scale bar = 1 cm. On top, close-up details of the rosette base in wild type and *TIE1* overexpression line. Schematic representations (left) and quantification (right) of rosette leaf axillary buds and branches of 40-day-old wild-type (B) and *35S-GFP-TIE1-19* plants (n = 8) (C). Left, arrows indicate buds (purple) and branches (blue). Right, bud and branch quantification in leaf axils is shown. Each box represents a rosette leaf node. Bud (purple), branch (blue), empty axil (grey). White boxes indicate that no leaf is formed at the position.

We previously identified a transcriptional repressor, TIE1, essential for the control of leaf development [25]. The gain-of-function *tie1-D* mutant, obtained by T-DNA activation-tagging, displays strong leaf developmental defects. We noticed that *tie1-D* also produced an excessive number of branches (S1 Fig), suggesting a possible role of *TIE1* in the control of shoot branching. To test this possibility, we first generated transgenic lines carrying the construct *35S-GFP-TIE1*, in which the *TIE1* coding sequence (CDS) fused to the *GREEN FLUORESCENT PROTEIN* (*GFP)* was driven by the Cauliflower Mosaic Virus 35S promoter (CaMV35S). The *35S-GFP-TIE1* plants displayed epinastic leaves as observed in *tie1-D* mutants (Fig 1A), which indicated that the GFP-TIE1 fusion protein was functional. We analyzed three *35S-GFP-TIE1* independent transgenic lines and found that all three lines produced more branches than the wild-type controls, and recapitulated the branching phenotype of *tie1-D* (Fig 1A–1C and S1 Fig). Because the homozygous *tie1-D* plants are sterile, we investigated the branching phenotype of plants of the fertile *35S-GFP-TIE1-19* line in detail. The results showed that although this *TIE1* overexpression line generated fewer rosette leaves than the wild-type plants (Fig 1A–1C), almost all the buds grew out to form branches, whereas in the wild-type controls most buds remained small at this stage (Fig 1B and 1C). These results suggest that *TIE1* is a positive regulator of axillary bud activity and shoot branching.

### Disruption of *TIE1* causes defects of shoot branching

**Fig 2:**
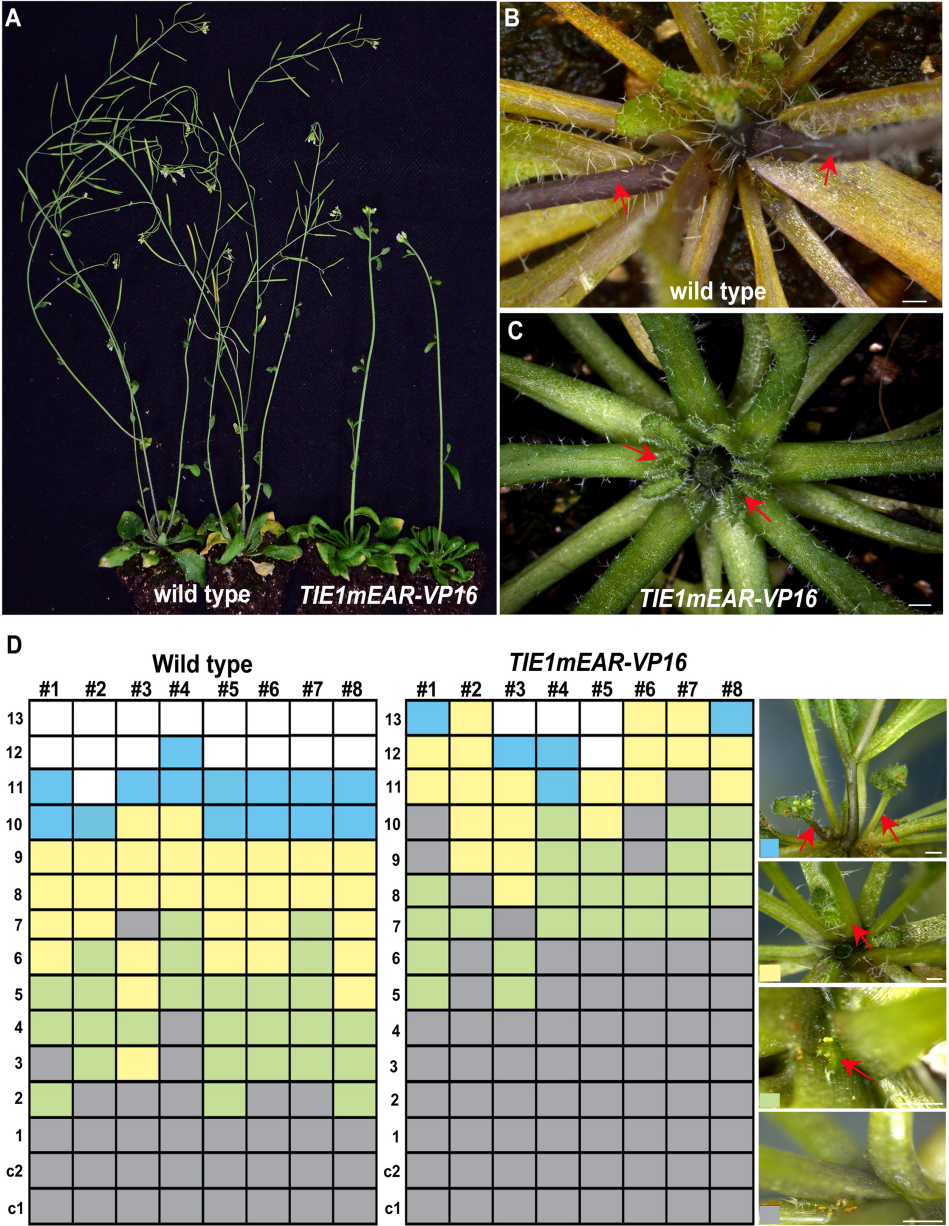
Disruption of *TIE1* leads to reduced shoot branching. (A) Expression of TIE1mEAR-VP16 chimeric protein causes defects in shoot branching. 45-day-old wild-type (left) and *TIE1mEAR-VP16* transgenic plants (right). Rosettes of wild-type (B) and *TIE1mEAR-VP16* plants (C). The primary inflorescence has been removed for better visualization of the axillary structures. Arrows point to branches (B) and buds (C). (D) Quantitative analysis of rosette axillary shoot development in 45-day-old wild-type and *TIE1mEAR-VP16* plants. The bud developmental stages were classified as follows: No bud: no axillary leaf primordia visible (grey); Small bud: axillary leaf primordia visible (green); Big bud: axillary leaf bearing many trichomes (yellow); Branch: visible elongating lateral inflorescence (blue). Representative images of these stages are shown in the right panels (from bottom to top). The white box indicates that no leaf is formed at the position. Scale bar = 1 mm.

*TIE1* belongs to a gene family with a high functional redundancy [25]. To overcome the difficulties caused by such genetic redundancy, we used a dominant-negative strategy to interfere with the function of all the TIE family members. We generated a *35S-TIE1mEAR-VP16* construct in which the TIE1 repressor was changed into an activator by mutating the EAR motif of TIE1 and fusing it to the VP16 activation domain. This approach has been successfully used to disrupt *TIE* genes redundancy in our previous study [25]. We examined all the rosette leaf axils of wild-type and *35S-TIE1mEAR-VP16* transgenic plants (Fig 2). Under the same growth conditions in which wild-type controls produced several branches, *TIE1mEAR-VP16* plants only had axillary buds but no branches (Fig 2A–2C). In addition, the degree of axillary bud development in the mutant and wild-type plants was classified into four classes, based on absence/presence of visible axillary leaf primordia and on axillary bud size (Fig 2D). The detailed analysis of the branching phenotypes showed that axillary bud development was obviously delayed in *TIE1mEAR-VP16* plants when compared to wild-type controls (Fig 2D): *TIE1mEAR-VP16* plants produced fewer branches than controls, and had more axils without a visible axillary bud. These results indicate that, like *BRC1*, *TIE1* regulates shoot branching from the early stages of axillary bud development.

### *TIE1* is expressed during axillary bud development

**Fig 3:**
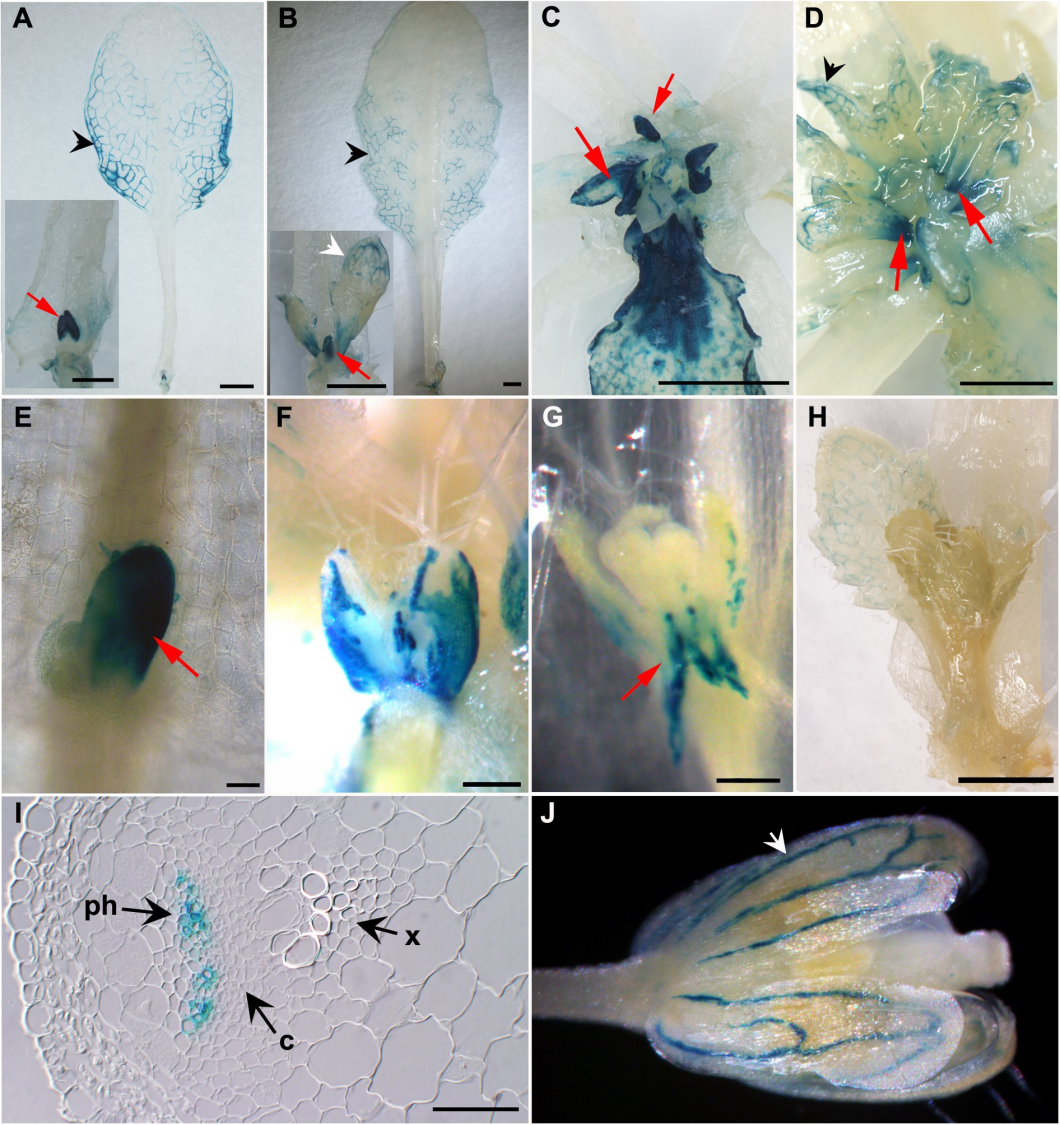
*TIE1* is expressed in the axillary buds. GUS histochemical activity of Arabidopsis *TIE1p*:*GUS*. Side view of young (A) and older (B) vegetative axillary buds (red arrows) and their subtending rosette leaves. Notice the GUS accumulation in young bud leaf primordia (red arrows), rosette leaf vasculature (black arrowheads) and the bud leaves (white arrowheads). Top view of the rosettes buds around the stem; young (C) and older (D) axillary buds (arrows) are shown. In older buds, GUS activity is restricted to the base of the bud. (E to H) close-up view of axillary buds of sequencial developmental stages. GUS signal becomes progressively restricted to the base of the bud (arrowhead in G). At the time of bud outgrowth the signal is no longer detectable (H). 3-mm transverse plastic-embedded section of a main stem showing GUS staining in phloem (ph) cells (I). x, xylem; c, cambium. (J) Close-up of a developing flower. Signal in the vasculature is indicated (white arrowhead). Scale bars = 1mm in (A) to (D), and (H); 50 μm in (E) and (I), 100 μm in (F) and (G).

To characterize in more detail the spatial and temporal expression patterns of *TIE1* during bud development, a 2790-bp genomic fragment upstream of the *TIE1* translation start codon was fused to the *β-GLUCURONIDASE* (*GUS*) reporter gene to generate a *TIE1pro-GUS* construct [25]. GUS staining analyses of the *TIE1pro-GUS* transgenic lines revealed that *TIE1* was predominantly expressed in developing axillary buds (Fig 3A–3G). In young axillary buds, signal was detectable throughout the leaf primordia (Fig 3A, 3C and 3E). As the buds developed, GUS signal became progressively more restricted to the base of the buds, and to the bud leaf vasculature (Fig 3B, 3D, 3F and 3G). When buds grew out into shoots, GUS activity was almost undetectable (Fig 3H). In addition, GUS accumulated in the stem vasculature, in particular in the phloem (Fig 3I), young leaf veins (Fig 3A and 3B) and sepal vasculature (Fig 3J). The expression patterns of *TIE1* during axillary bud development resemble those of *BRC1* [6]. These results are consistent with an important role of *TIE1* in the control of axillary bud activity.

### TIE1 interacts with the transcription factor BRC1

**Fig 4:**
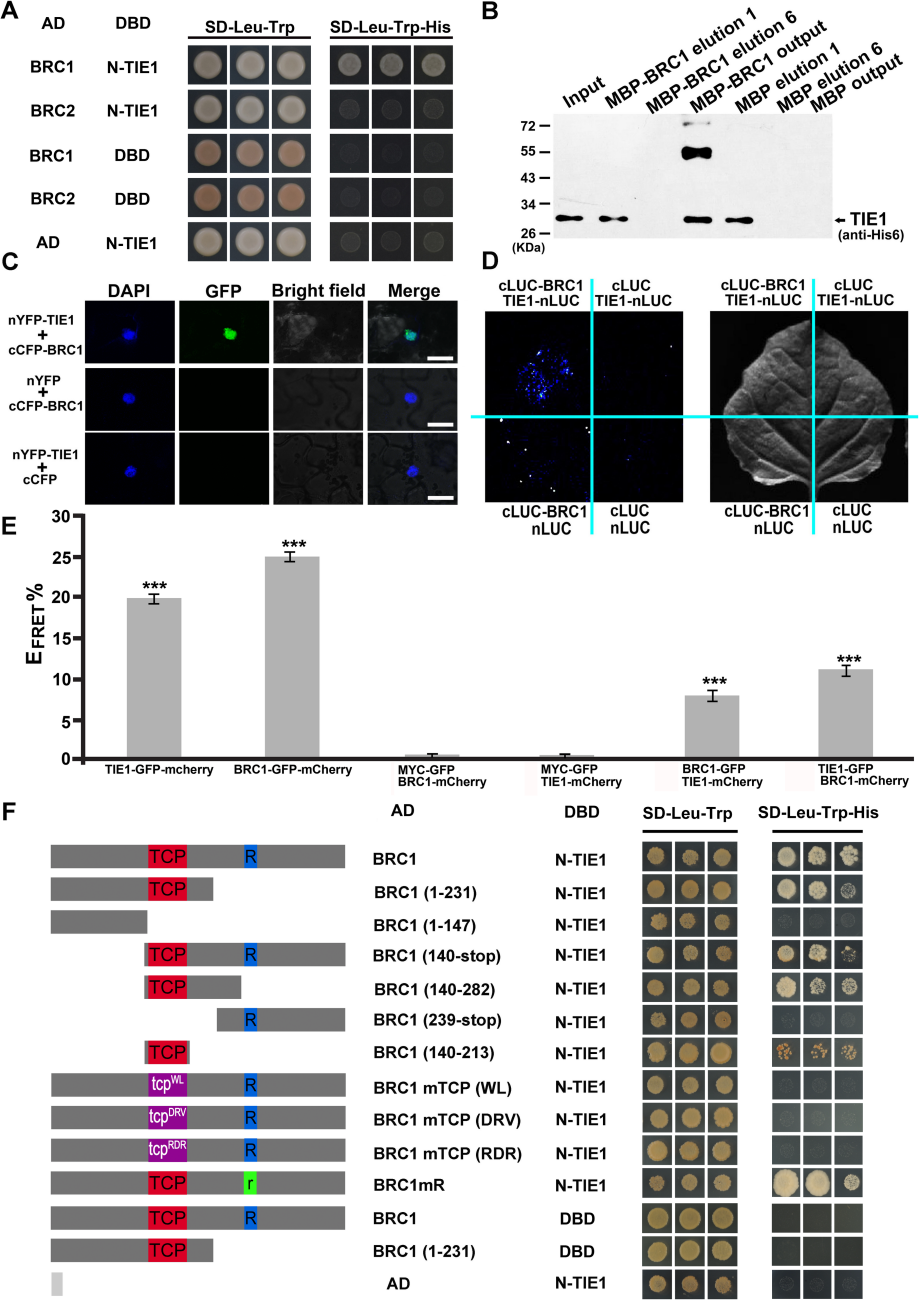
TIE1 interacts with BRC1 *in vitro* and *in vivo*. (A) Yeast two-hybrid assays of TIE1 with BRC1 and BRC2. The N-t of TIE1 (1 to 108 residues) was fused to GAL4–DBD to generate the bait protein. The transcription factors BRC1 and BRC2 were fused with the GAL4–AD to generate the prey protein. AD, activation domain; DBD, DNA binding domain. Co-transformed yeast cells were grown on medium lacking Leu and Trp (SD-Leu-Trp) and selected on medium lacking Leu, Trp and His (SD-Leu-Trp-His) with 5 mM 3-amino-1,2,4-triazole (3-AT). The empty vectors *PDEST32* or *PDEST22* were used as negative controls. The three spots are triplicates of the experiment. (B) Pull-down assays to test TIE1 interaction with BRC1 *in vitro*. The MBP-BRC1 was used to pull down TIE1-His with the MBP as a negative control. The mixtures MBP-BRC1/TIE1-His and MBP/TIE1-His were incubated with Amylose resin (NEB) and the beads were washed six times to avoid the false positive. The “elution 1” and “elution 6” mean the supernatant in the first wash and sixth wash of the MBP-BRC1/TIE1-His and MBP/TIE1-His beads, respectively. In the sixth wash, the TIE1-His was not detected in the supernatant, indicating the amylose resin was cleaned up and avoiding the false positive. After the six washes, the bound proteins in the beads (precipitate) were eluted with 2×SDS buffer and detected with anti-His6. The TIE1-His could be pulled down by MBP-BRC1 (the lane designated MBP-BRC1 output) but not by MBP control (the lane designated MBP output). (C) BiFC assay of the interaction between TIE1 and BRC1. GFP signal was observed in *N*. *benthamiana* leaves co-transformed with *nYFP-TIE1* and *cCFP-BRC1* vectors. *cCFP-BRC1*/*empty nYFP* and *nYFP-TIE1*/*empty cCFP* were used as negative controls. (D) Interaction between TIE1 and BRC1 *in vivo* tested by firefly LUC complementation imaging (LCI) assay. The signal of LUC activity was detected in the *cLUC-BRC1*/*TIE1-nLUC* combination. *cLUC/TIE1-nLUC*, *cLUC-BRC1*/*nLUC* and *nLUC*/*cLUC* were used as negative controls (E) *In vivo* confirmation of TIE1-BRC1 interaction by FRET-acceptor photobleaching assay. E-_FRET_% is calculated as relative increase in GFP fluorescence intensity after photobleaching of the mCherry acceptor. Intramolecular FRET (both fluorophores were in the same protein, TIE1-GFP-mCherry; BRC1-GFP-mCherry) and background signal measured using a non-interacting protein (epitope MYC-GFP) are included as positive and negative controls, respectively. Results show mean ± SEM (n = 10 cells from at least 3 different leaves); E_FRET_% values obtained for the BRC1-GFP/TIE1-mCherry and TIE1-GFP/BRC1-mCherry interaction are significantly different from values obtained for both negative controls (MYC-G/BRC1-C and MYC-G/TIE1-C). *** p-value<0.001 in a two-tailed Student’s t-test. (F) TIE1 interacts with the TCP domain in yeast two-hybrid assays. Left, schematic representation of the different BRC1 deletions and BRC1 mutant proteins (in the TCP domain: tcp^WL^, tcp^DRV^, tcp^RDR^ or in the R domain: BRC1mR) that were fused to the GAL4 prey (AD). Yeast two-hybrid assays were carried out between these BRC1-AD proteins and an N-t of TIE1 (1 to 108 residues) fused to GAL4–DBD bait. The empty vectors *pGADT7* or *pGBKT7* were used as negative controls. The three spots are triplicates of the experiment.

To investigate the molecular mechanisms by which *TIE1* regulates shoot branching, we performed a yeast two-hybrid screening of an Arabidopsis transcription factor library to identify TIE1 interactors, using as a bait of a protein containing the N-terminal (N-t) 108 amino acid residues of TIE1 [25]. The results showed that the N-t region of TIE1 interacted with BRC1 but not with BRC2. We therefore cloned the CDS of Arabidopsis *BRC1* and *BRC2* to verify this interaction. The yeast-two-hybrid assays confirmed that BRC1 interacted with TIE1, whereas BRC2 did not (Fig 4A). We then performed additional experiments to further investigate BRC1-TIE1 interaction *in vitro* and *in vivo*. First, we expressed BRC1 fused to the MALTOSE BINDING PROTEIN (MBP-BRC1) and His-tagged TIE1 in *Escherichia coli* and purified them for *in vitro* pull-down assays. Specific binding of His-tagged TIE1 was detected in the MBP-BRC1 after eluting six times, while no band of His-tagged TIE1 was observed in the control MBP, indicating that TIE1 interacted strongly with BRC1 *in vitro* (Fig 4B). Then, we confirmed the association of BRC1 with TIE1 *in vivo* by BiFC and firefly luciferase complementation imaging assays (Fig 4C and 4D). Finally, BRC1 and TIE1 interaction was further confirmed by acceptor photobleaching fluorescence resonance energy transfer (APB-FRET) using transient assays in *Nicotiana benthamiana* leaves (Fig 4E).

BRC1 contains several important domains including a TCP domain for dimerization and DNA binding, and an R domain of unknown function [27]. To map the regions of the BRC1 protein necessary for interaction with TIE1, we generated a series of BRC1 deletions lacking different regions of the protein (Fig 4F). Yeast two-hybrid assays showed that the TCP domain was necessary and sufficient for the interaction between TIE1 and BRC1, whereas the R domain was not required for the interaction. We further assayed three BRC1 proteins with point mutations in the TCP domain, two in the basic region and one in helix II. All three mutations disrupted the TIE1-BRC1 interaction (Fig 4F), whereas a point mutation in the R domain did not affect the interaction. These data demonstrate that BRC1 interacts with the N-t region of TIE1 through its TCP domain. These results together with the observed overlapping expression patterns of *BRC1* and *TIE1* in axillary buds support the existence of this interaction *in planta*.

We further examined whether other members of the TIE family could interact with BRC1. Yeast two-hybrid analyses showed that TIE2 and TIE4 also interacted with BRC1, while TIE3 did not (S2 Fig). These results suggest that several members of the TIE protein family may control shoot branching by directly interacting with BRC1.

### TIE1 represses BRC1 activity

**Fig 5:**
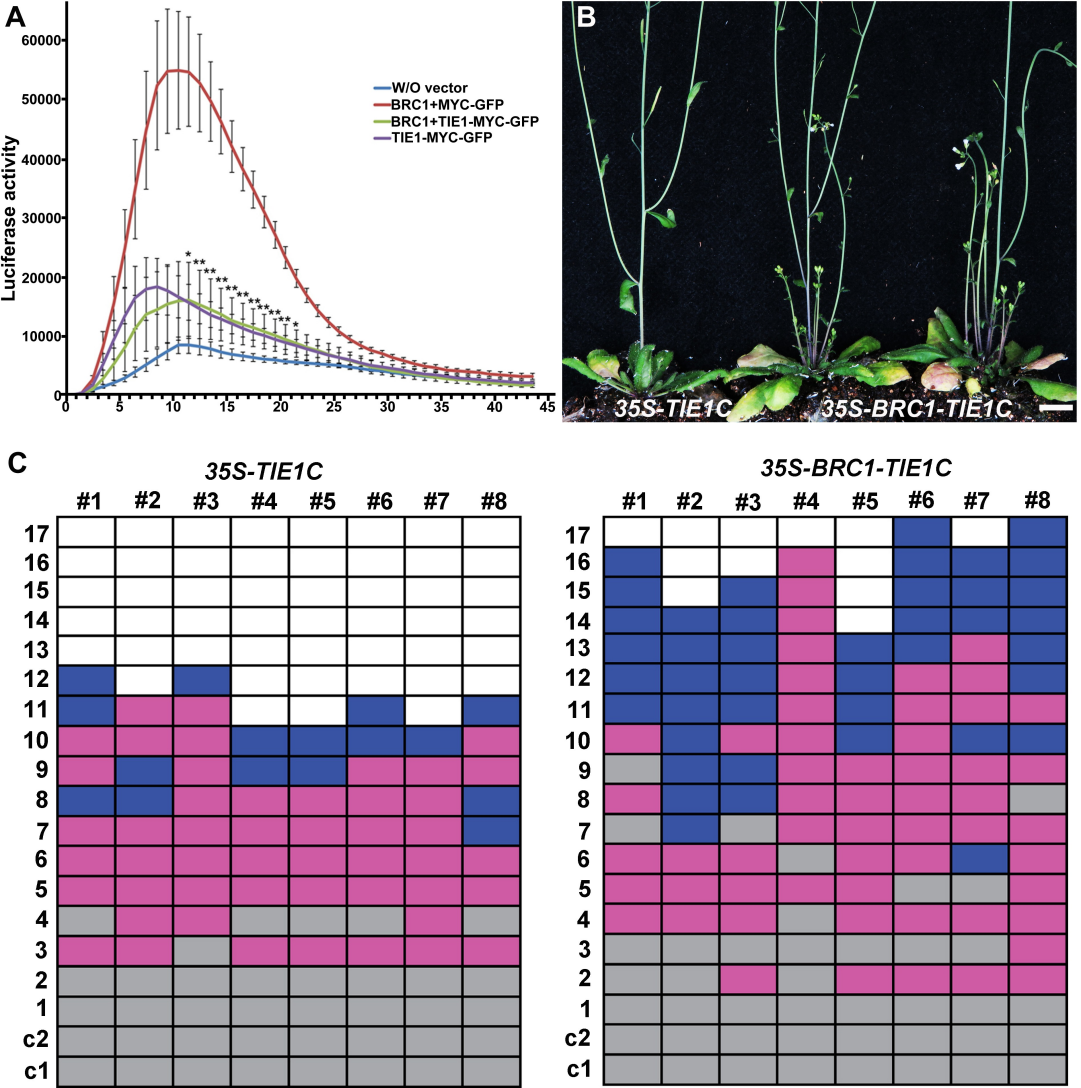
TIE1 inhibits BRC1 transcriptional activity. (A) A 2kb-long genomic region upstream of *HB53* gene fused to *LUC* was used as a reporter for the transactivation assay. Results are represented as mean ± SEM (n = 6). Co-infiltration of *BRC1* with *TIE1* (light green line) considerably reduced the activation of the promoter as compared to the co-infiltration with the MYC-GFP epitope (red line; *** p <0.005; ** p <0.01 in a two-tailed Student’s T test). (B) Phenotype of 45-day-old transgenic plants expressing a chimeric *BRC1-TIE1C* (right) or a *35S-TIE1C* control (left). Scale bar = 1 cm. (C) Quantification of buds and branches in the rosette leaf axils of 40-day-old *35S-TIE1C* and *35S- BRC1-TIE1C* plants (n = 8). Each box represents a rosette leaf node. Purple box indicate bud; blue, branch; grey, empty axil; white, no leaf formed at the position.

We then examined whether BRC1 transcriptional activity could be regulated by interaction with the transcriptional repressor TIE1. For that we used the reporter construct *HB53pro-LUC*, in which the *LUCIFERASE* (*LUC*) gene is driven by a 2000 bp promoter region of *HB53*, which is a BRC1 direct target gene [18]. We co-infiltrated *Nicotiana benthamiana* leaves with *HB53pro-LUC* and *35S-BRC1* (and a *35S-MYC-GFP* control). *LUC* activity analysis indicated that BRC1 was able to activate directly the *HB53pro-LUC*. In contrast when *HB53pro-LUC* and *35S-BRC1* were co-infiltrated with *35S-TIE1-MYC-GFP*, the activation of *HB53pro-LUC* was very much reduced (Fig 5A), suggesting that TIE1 inhibits BRC1 transcriptional activity. We have shown that TIE1 serves as a bridge between TCPs (with its N-t region) and the corepressors TPL/TPRs (with its C-t region) [25]. To further examine the effects of the TIE1-BRC1 interaction, we generated a fusion protein BRC1-TIE1C in which BRC1 was physically linked with the C-t of TIE1 (from the 108^th^ amino acid residue to the stop codon). The fusion protein was expressed in wild-type plants using a CaMV 35S promoter. The detailed branching phenotypes of these transgenic plants were analyzed (Fig 5B and 5C). The *35S-BRC1-TIE1C* transgenic lines produced about six branches, whereas the control *35S-TIE1C* transgenic plants produced two to three branches under the same growth conditions (Fig 5B and 5C). The increased branching of 35S-BRC1-TIE1C transgenic lines indicated that BRC1 protein activity was affected by the fusion with TIE1, probably due to the recruitment of TPL/TPRs corepressor by TIE1 C-t.

### *TIE1* regulates genes involved in shoot branching

**Fig 6:**
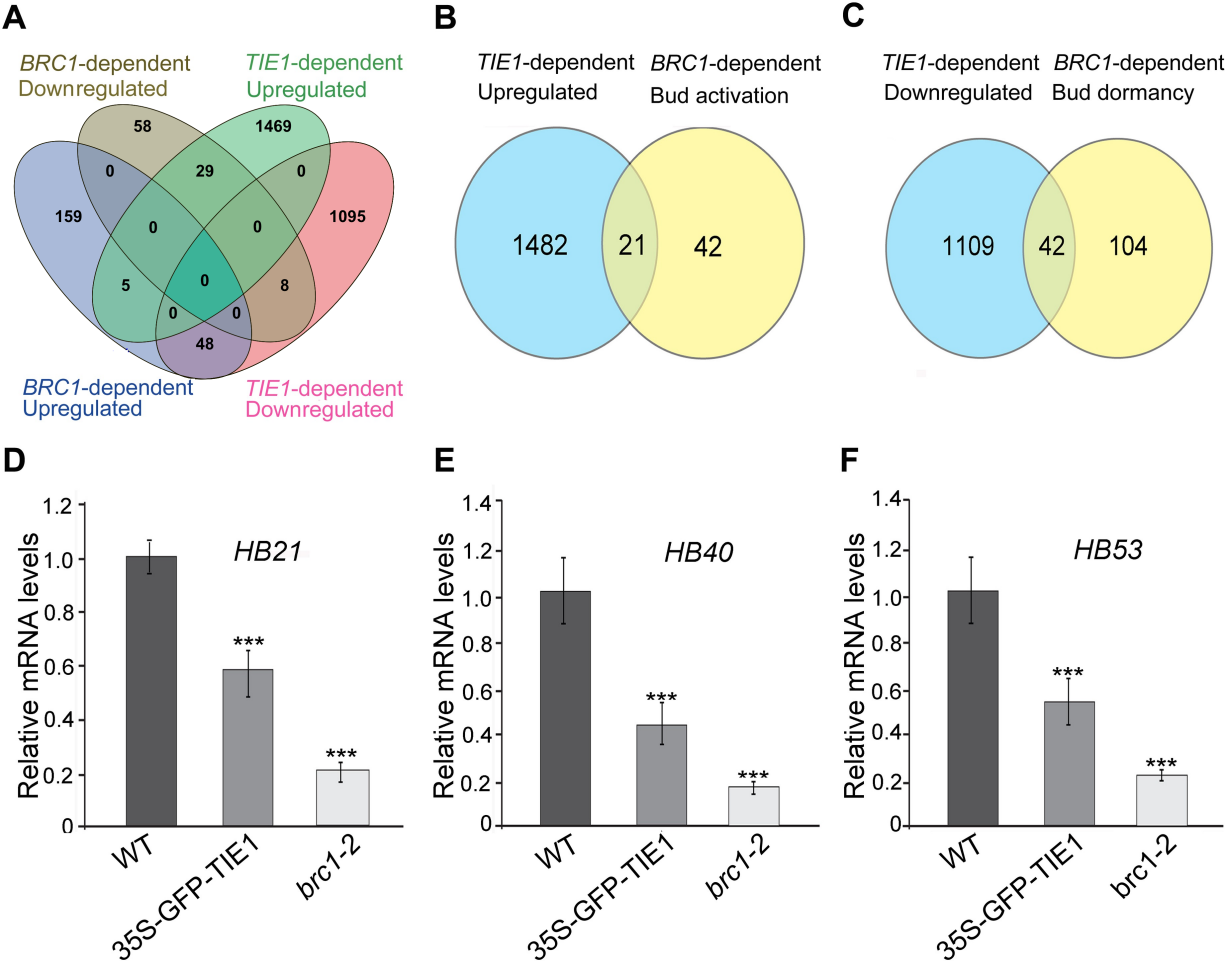
TIE1 regulates *BRC1*-dependent genes involved in bud development. (A) to (C) Venn diagrams showing significant overlap between *TIE1*-regulated genes and *BRC1*-dependent genes. Detailed gene information is shown in S1–S3 Tables. (D) to (F) mRNA levels of *HB21*, *HB40* and *HB53* were quantified by qRT-PCR in wild type, *35S-GFP-TIE1* plants and *brc1-2* mutants. The expression was normalized to *AtUBQ10* levels, and was relative to wild-type levels. Data represents mean ± SD from three biological replicates. Significant differences are indicated ***P < 0.001 (two-tailed Student’s t-test).

To further elucidate the mechanisms by which *TIE1* regulates shoot branching, we performed RNA-seq transcriptome analysis of rosette leaf axil tissue (highly enriched in axillary buds) of *35S-GFP-TIE1* overexpression lines and wild-type controls. We found that 1503 genes were upregulated and 1151 genes downregulated in the *TIE1* overexpression line (q value<0.05; fold change≥1.5 and ≤-1.5) (S1 Table). We compared these genes with a list of 307 *BRC1*-dependent genes (False Discover Rate <0.05) [20], and found a negative correlation between *TIE1*-responding genes and the *BRC1*-dependent genes: *TIE1*-upregulated genes appeared at a much higher frequency than expected for a random gene list among the *BRC1*-downregulated genes (30% of the *BRC1*-downregulated genes, p value = 2.1E-18 in a hypergeometric test) (Fig 6A and S2 Table). Likewise *TIE1*-downregulated genes were significantly enriched among the *BRC1*-upregulated genes (22% of the *BRC1*-upregulated genes, p value = 1.5E-27 in a hypergeometric test) (Fig 6A and S2 Table), supporting that *TIE1* acts to antagonize *BRC1* activity during bud development. These enrichments were remarkably higher than those found when the comparison was done between *TIE1*-dependent genes and the *BRC1*-independent genes obtained in the same experiment (i.e. genes that respond to a low Red:Far red light ratio, both in wild type and *brc1* mutants) [20].

Then we further compared *TIE1*-dependent genes with a particular subset of *BRC1*-dependent genes that also responded significantly to decapitation [28]. Genes upregulated in response to *BRC1* and downregulated 24 hours after decapitation were termed *Bud dormancy genes*. Genes downregulated in response to *BRC1* and upregulated 24 hours after decapitation were termed *Bud activation genes* [20]. Again, *TIE1*-dependent upregulated genes appeared among *Bud activation genes* at a much higher frequency than that expected in a random gene list (33%, p value = 6.55E-15) and *TIE1*-dependent downregulated genes were significantly enriched among *Bud dormancy genes* (29%, p value = 5.29–29).

Interestingly, three *BRC1* direct targets, *HB21*, *HB40* and *HB53* [18], were among the *TIE1*-downregulated genes (Fig 6C, S2 and S3 Tables). Quantitative RT-PCR analysis confirmed that the transcriptional levels of these genes were significantly lower in the *TIE1* overexpression line and in *brc1-2* mutants than in wild-type controls (Fig 6D and 6F). These data indicate that TIE1 modulates the expression of sets of BRC1-dependent, bud activation and dormancy genes.

Finally, we also found that in our RNA-seq data *BRC1* was downregulated (S3A Fig), and qRT-PCR analyses confirmed that *BRC1* mRNA levels were much lower in *TIE1* overexpression lines than in the wild-type control (S3B Fig). Conversely, *BRC1* expression levels were significantly higher in three *35S*:*TIE1mEAR-VP16* lines than in the wild-type control (S3C Fig). These results suggest that *TIE1* may also directly or indirectly regulate *BRC1* at the transcriptional level.

## Discussion

**Fig 7:**
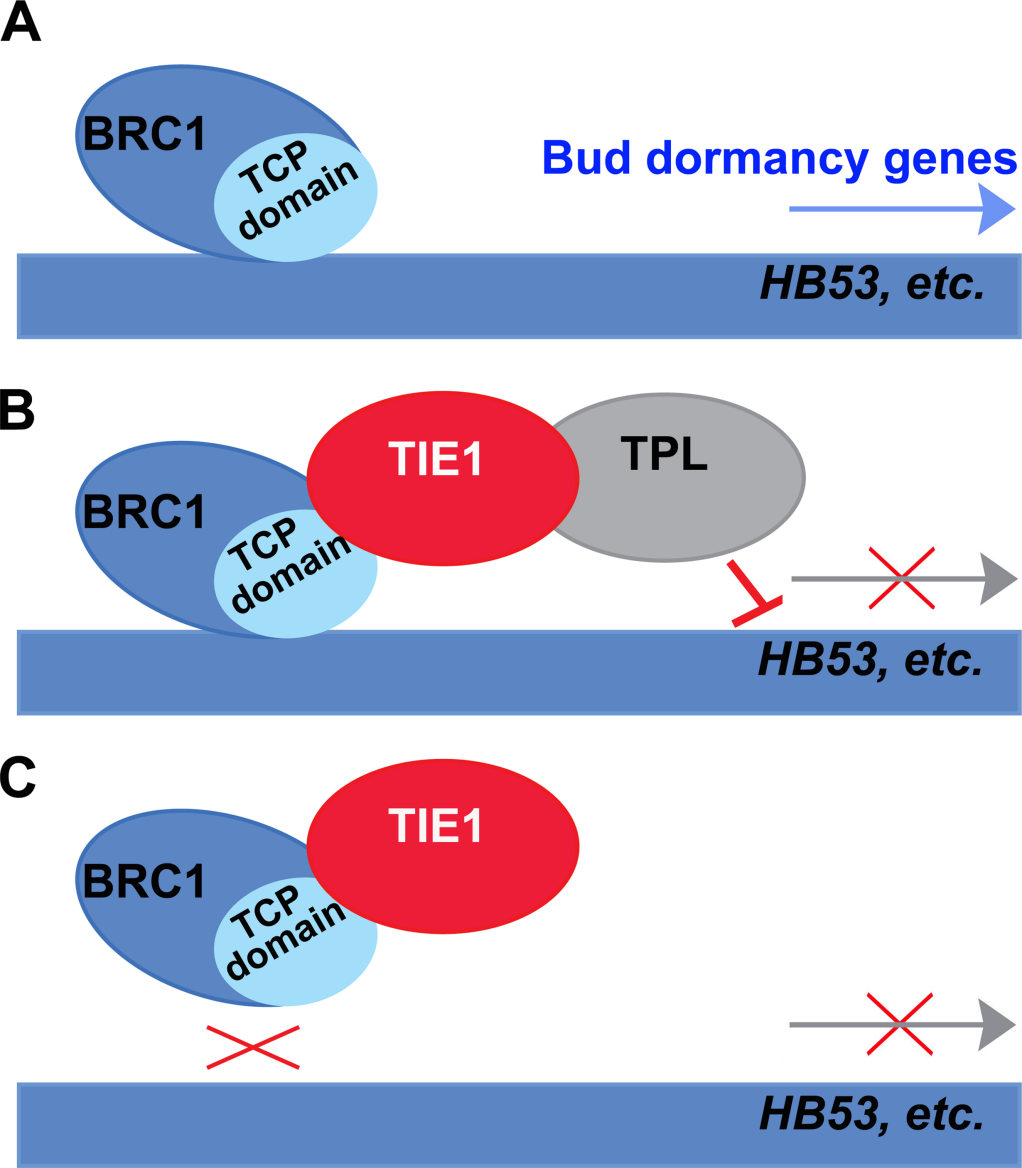
Model for *TIE1* function in the *BRC1*-dependent regulation of shoot branching. (A) In the absence of TIE1, BRC1 promotes the expression of bud dormancy genes such as *HB53* and inhibits shoot branching. (B) and (C) When TIE1 binds BRC1, TIE1 recruitment of TPL/TPR co-repressors [25] leads to transcriptional repression of BRC1 targets (B) or TIE1 sequesters and prevents BRC1 binding to the promoters of target genes (C). Bud dormancy genes such as *HB53* are downregulated and shoot branching is promoted.

In this study, we discovered that the transcriptional repressor TIE1 is a regulator of shoot branching. The gain-of-function mutant *tie1-D* and transgenic plants overexpressing *TIE1* produce more branches, whereas *TIE1* loss-of function leads to lower bud activity and fewer branches. In addition, we demonstrated that TIE1 interacts, *in vitro* and *in vivo*, with BRC1, a transcription factor that plays an important role in the control of bud activity. Furthermore, *TIE1* is expressed in young axillary buds and is regulated during bud development in patterns that overlap with those of *BRC1* [6], further supporting the possibility that TIE1 interacts with BRC1 *in planta*. By binding to BRC1, TIE1 inhibits BRC1 activity and consequently represses the transcription of many BRC1 target genes (Fig 7A and 7B). Our data not only demonstrate that TIE1 is an important regulator in the control of shoot branching, but also provides evidence of a novel layer of regulation of BRC1 at the protein level.

TCP proteins are plant-specific transcription factors that group into class I and class II subclasses on the basis of sequence similarity [29,30]. The class II TCPs are further categorized into CINCINNATA-like TCPs and CYCLOIDEA/TB1 (CYC/TB1)-like TCPs [30]. The modulation of TCP activity at the protein level is important for plant development. Some proteins, including the SWI/SNF chromatin remodeling ATPase BRAHMA (BRM) and the ARMADILLO BTB ARABIDOPSIS PROTEIN1 (ABAP1) have been reported to interact with CIN-like TCPs and regulate their activity [31,32]. Recently, we found that the EAR-motif containing repressor TIE1 suppresses CIN-like TCP activity by recruiting the transcriptional co-repressors TOPLESS (TPL)/TOPLESS-RELATED (TPR) proteins during leaf development [25]. A yeast two-hybrid screening revealed that TIE1 also interacts with BRC1 (a TCP factor of the CYC/TB1 subclade), a prominent bud-specific regulator of shoot branching [6,10]. However, the role of TIE1 in the control of shoot branching had not been identified so far. In this paper, our genetic and biochemical data indicate that TIE1 is not only an important factor regulating CIN-like TCPs, but also regulates BRC1 at the protein level. TIE1 interacts with BRC1 and prevents the transcription of BRC1 target genes. Like in the case of CIN-like TCPs, TPL/TPRs could be recruited by TIE1 to repress BRC1 activity during bud development (Fig 7A and 7B). This is consistent with the observation that plants expressing a BRC1 fusion protein carrying the C-terminal of TIE1 produce more branches.

Interestingly, other TPL/TPR-interacting proteins have been previously implicated in the control of shoot branching through SL signaling. Indeed, the rice SL signaling repressor D53, and its orthologs in Arabidopsis, SMXL6, SMXL7 and SMXL8, interact with TPL/TPR proteins [33–36]. This interaction may promote TPL/TPR oligomerization and formation of a repressor-corepressor nucleosome complex [37]. This interaction has been proposed to be responsible for the transcriptional repression of *OsTB1*/*BRC1* although this is yet unclear [38,39]. Our findings show that TPL/TPRs are also recruited by TIE1 to directly repress BRC1 at the protein level, suggesting that the TPL/TPRs use different molecular mechanisms to control shoot branching. Furthermore, rice D53 interacts with Ideal Plant Architecture 1 (IPA1), another negative regulator of shoot branching [40–42] that binds the *OsTB1/FINE CULM1* promoter [22], and may affect its expression. This interaction, conserved in wheat, leads to suppression of the transcriptional activity of IPA1-like factors [42]. These observations indicate that TPL/TPRs-interacting proteins, such as TIE1-like and D53-like proteins, play important roles in the control of shoot branching both in dicots and monocots. The rice genome has six TIE1 homologs [43,44]. It will be very interesting to determine whether OsTIEs interact with OsTB1/FINE CULM1 and TPL/TPR corepressors.

Remarkably, our deletion and mutation analysis suggested that TIE1 interacts with the TCP domain of BRC1, responsible for DNA binding [30]. The TCP domain of BRC1 is necessary and sufficient for the TIE1 and BRC1 interaction, because point mutations in the TCP domain completely abolish this interaction. This raises the alternative possibility that TIE1 represses BRC1 activity by preventing BRC1 binding to DNA as it has been described for DELLA proteins, which repress the activity of TCP14 and TCP15 by interacting with their TCP domains [45]. Therefore, TIE1 is likely to inhibit BRC1 activity either by recruiting the transcriptional repressor machinery and/or by hindering the TCP domain of BRC1 from binding the promoters of target genes (Fig 7B and 7C). These two mechanisms may work together to precisely regulate BRC1 activity and shoot branching in response to internal and external cues. In addition, we found that *BRC1* itself was down-regulated by TIE1, which indicates that TIE1 may also control directly or indirectly *BRC1* at the transcriptional level. Investigating how this transcriptional and post-transcriptional regulation of BRC1 by TIE1 affects plant architecture remains to be determined.

Recently, ABA has been reported to negatively regulate axillary bud growth in Arabidopsis [46]. BRC1 is an important regulator of ABA signaling in buds partly through the regulation of three genes, *HB21*, *HB40* and *HB53*, encoding HD-ZIP transcription factors [18]. Our results showed that TIE1 regulates about one third of the *BRC1*-dependent genes induced in dormant buds including *HB21*, *HB40* and *HB53*, which raises the possibility that TIE1 helps BRC1 finely tune the transcriptional level of these branching control genes.

It is worth noting that the phenotype of *TIE1* gain-of-function plants is not identical to that of *brc1* mutants: in addition to an excess of branching, the former display many other phenotypes including epinastic leaves, dwarfism and early flowering [25]. Indeed, *TIE1* expression in tissues other than axillary buds (e.g. leaf, sepal and stem vasculature) as well as the reported interaction of TIE1 with other transcription factors and TPL/TPRs (see above) may account for these phenotypes unrelated to shoot branching of *TIE1* gain-of-function lines.

We have recently found that TIE1 is ubiquitinated by several E3-ligase proteins TEARs (**T**I**E**1-**a**ssociated **R**ING-type E3 ligase**s**) for degradation [26]. Interestingly, disruption of TEARs using the dominant-negative strategy and sextuple *tear* mutants also cause excessive branching [26], this is consistent with our observation in this study that overexpression of *TIE1* promotes shoot branching. The characterization of potential additional components of the molecular machinery that controls shoot branching via modulation of the activity of the BRC1 protein will help us further understand the complex regulatory mechanisms that determine plant shoot architecture in response to environmental cues.

## Materials and methods

### Plant materials and growth conditions

The *Arabidopsis thaliana* ecotype Columbia-0 (Col-0) was used in this study. The mutants *tie1-D* and *brc1-2* were described previously [6, 25]. Half-strength Murashige and Skoog medium with or without 20 μg/mL DL-phosphinothricin or 50 μg/mL kanamycin were used for growing or screening the plant seeds. The plates with seeds were placed at 4°C for 2 d synchronization before being incubated at 22°C under long-day conditions (16-h light and 8-h dark, 70% relative humidity). The seven-day-old seedlings were transferred to soil and were grown under the same conditions as described above.

### Generation of binary constructs and transformation

To generate the *TIE1* overexpression line, the *TIE1* coding sequence was amplified from Arabidopsis seedling cDNA using the primer pairs TIE1-F/R (S4 Table). The PCR product was cloned into pENTR/D TOPO (Invitrogen) to generate pENTR-TIE1. Then, the overexpression construct *35S-GFP-TIE1* was generated by an LR reaction between pENTR-TIE1 and pB7GWF2 (Ghent University). To examine the temporal and spatial expression pattern of TIE1, the 2790-bp genomic fragment upstream of TIE1 start codon was amplified using the primers TIE1P-F and TIE1P-R and was cloned into pENTR/D-TOPO to generate pENTR/D-TIE1P. TIE1P-GUS was generated by LR reaction between pENTR/D-pTIE1 and pKGWFS7 (Ghent University). To generate *35S-BRC1-TIE1C* construct, the coding region of BRC1 without a stop codon was amplified from Arabidopsis seedling cDNA with primers BRC1-F1/R1 and further was cloned into pENTRY/D-TOPO to generate pENTRY-BRC1N. The CaMV 35S promoter was amplified from vector pWM101 with primers p35S-F/R. The fragment was cloned into pDONRP4P1r (Invitrogen) to generate pENTRY-L4-35S-R1. The C-t of TIE1 sequence was amplified from pENTR-TIE1 using primers TIE1C-F/R and was cloned into pDONRP2rP3 (Invitrogen) to generate pENTR-R2-TIE1C-L3. The 35S-BRC1-TIE1C construct was generated by LR reaction from pENTRY-L4-35S-R1, pENTRY-BRC1N, and pENTR-R2-TIE1C-L3 and pK7m34GW (Ghent University). To generate *35S-TIE1mEAR-VP16*, TIE1mEAR was amplified with primers TIE1m-F/R and the PCR product was cloned into NTRY/D-TOPO to generate pENTRY-TIE1mEAR. The coding region of VP16 was amplified from pTA7002 [47] and was cloned into pQDR2L3 with primers VP16-F/R to generate pENTR-R2-VP16-L3. The 35S-TIE1mEAR-VP16 construct was generated by LR reactions among plasmids pK7m34GW, pENTRY-L4-35S-R1, pENTRY-TIE1mEAR and pENTR-R2-VP16-L3. These constructs were transformed into *Agrobacterium tumefaciens* GV3101/pMP90 by electroporation method and then into Arabidopsis as described previously by floral dip method [48].

### Histochemical GUS staining

For GUS staining, tissues from *TIE1pro-GUS* lines were soaked in 90% acetone solution for 20 mins on the ice and washed by phosphate buffer twice. Then the samples were incubated in GUS staining buffer containing 0.5 mg/mL 5-bromo-4-chloro-3-indolyl glucuronide and vacuumed for 30 min before incubation overnight at 37°C. The staining buffer was then replaced by 70% ethanol for decolorizing before microscopy analysis. Plastic embedding and sectioning of GUS-stained stem fragments of adult plants was carried as described in Chevalier et al. [49].

### Gene expression assays

For quantitative RT-PCR, total RNAs of the tissues around leaf axils from 25-day-old wild-type, *35S-GFP-TIE1* and *brc1-2* were extracted using TRIzol reagent (Invitrogen). Reverse transcription was carried out using Superscript II Reverse Transcriptase Kit (Invitrogen). Quantitative RT-PCR was performed with three biological repeats using SYBR Green Realtime PCR Master Mix (Toyobo) and using the diluted cDNA as the template. The 2^-ΔΔCT^ method was used to calculate the relative expression level of each gene [50]. Primers used were listed in S4 Table. *AtUBQ10* gene was used as an internal control.

For RNA-seq, total RNAs of the tissue surrounding leaf axils enriched in axillary buds from 25-day-old wild-type and 35S-GFP-TIE1 plants were extracted using TRIzol reagent. The RNA-seq was performed on the Illumina HiSeq 2000 platform (Illumina) at the Biodynamic Optical Imaging Center (BIOPIC) of Peking University. The bioinformatic and statistical analysis of the RNA-seq data was performed according to the procedures described previously [51]. Genes with changes of more than 1.5-fold (Q-value ≤ 0.05) were defined as differentially expressed genes. The hypergeometric test was performed in the software environment R (CRAN) using the phyper function.

### Yeast two-hybrid assays

To test the interaction between TIE1 and BRC1/BRC2, the N-t of TIE1 (1–108) and the coding sequences of BRC1/BRC2 were amplified with primers TIE1N-F/R and BRC1-F/R or BRC2-F/R listed in S4 Table. The products were cloned into pENTR/D-TOPO to generate pENTRY-TIE1N and pENTRY-BRC1/BRC2. The bait construct DBD-TIE1N was generated by LR reaction between pDEST32 (Invitrogen) and pENTRY-TIE1N. The prey construct AD-BRC1/BRC2 were generated by LR reaction between pENTRY-BRC1/BRC2 and pDEST22 (Invitrogen). The bait construct and each prey one were co-transformed into the yeast strain AH109. Medium without Leu, Trp and His and with 5 mM 3-amino-1,2,4-triazole (3-AT) was used for selection.

To determine which region of BRC1 interacts with TIE1, fragments of BRC1 where amplified by PCR and cloned in pDONR207 by BP clonase (Thermofisher) and then inserted in pGADT7-GW by LR recombination using Gateway LR clonase II (Thermofisher). The TIE truncated in the C-t part (TIE1(1–108)) was also cloned in pDONR207 and inserted afterwards in pGBKT7-GW (YTH assays; Thermofisher). Vectors were transformed in yeast strain AH109 and medium without Leu, Trp and His and with 5 mM 3-amino-1,2,4-triazole (3-AT) was used for selection.

### Pull-Down assays

The MBP-BRC1 construct was generated by LR reaction between pENTR/D-BRC1 and pMAL-GW modified from pK2GW7 (Ghent University). The pET28-TIE1-His construct was generated by enzyme digestion reaction with the *Eco*R I and *Sal* I sites of pET-28a (+) (Novagen). The constructs were introduced into *E*. *coli* BL21 (DE3) competent cells for protein expression. The transformed cells were cultured in LB medium at 37°C until the OD600 reached 0.5 and then moved to 18°C condition for 12h in the presence of 0.5mM IPTG for the induction of protein expression. The proteins were extracted in buffer containing 20 mM Tris-HCl [pH7.4], 200 mM NaCl, 1 mM EDTA, 1 mM PMSF, and 1×C-complete protease inhibitor [Roche]. Bacterial lysates in extraction buffer contained 50 μg MBP-BRC1 or the control MBP proteins were mixed with lysates containing 50 μg TIE1-His fusion protein. The mixtures were incubated with Amylose resin (NEB) at 4°C for 3 h. Beads were washed six times with the column buffer (20 mM Tris-HCl, pH 7.4, 200 mM NaCl, 1mM EDTA, 1mM PMSF, and 1×C-complete protease inhibitor [Roche]). The supernatant in the first wash (elution 1) and sixth wash (elution 6) of the beads was boiled with 2×SDS buffer for further immunoblot analysis. After the sixth washes, the bound proteins (precipitate) were eluted with 2×SDS buffer and boiled for 5 min. Immunoblot analysis was then performed to detect the proteins with anti-His antibody (Sigma-Aldrich).

### BiFC assays and LCI assay

The constructs *nYFP-TIE1* and *cCFP-BRC1* were generated by LR reactions between *pENTR-TIE1* and *pnYFPXGW* or between *pENTR-BRC1* and *pcCFPXGW* [52]. The above constructs were first transformed into *A*. *tumefaciens* GV3101 and then the *nYFP-TIE1* and *cCFP-BRC1/BRC2* were co-infiltrated into the leaves of *N*. *benthamiana*. The plants were grown in the dark for 12 h followed by 48 h in a growth chamber under normal conditions. The fluorescence signal of GFP in *N*. *benthamiana* leaf cells was observed under a Leica SPE confocal microscope (Leica). A DAPI (Sigma) solution was used to stain the nuclei. The excitation laser was set at 488 nm for GFP and 405 nm for DAPI staining. For LCI assay, the constructs *TIE1-nLUC* and *cLUC-BRC1* were generated by cloning the *TIE1* gene into *pCAMBIA-nLUC-GW* and by cloning *BRC1* gene into *pCAMBIA-cLUC-GW* [26]. The above constructs were first transformed into *A*. *tumefaciens* GV3101 and then the different combinations of the constructs, i.e. *cLUC-BRC1* and *TIE1-nLUC*, *cLUC* and *TIE1-nLUC*, *cLUC-BRC1* and *nLUC*, were co-infiltrated into the *N*. *benthamiana* leaves. The plants were placed in the dark for 12 h followed by 48 h in a growth chamber under normal condition. The infiltrated *N*. *benthamiana* leaves were sprayed with luciferin (100 mM) and kept in dark for 10 mins. The leaves were observed under a low-light cooled charge-coupled device (CCD) imaging apparatus Lumazone_1300B (Roper Bioscience).

### Acceptor photo-bleaching fluorescence resonance energy transfer (APB- FRET)

The *TIE1* and *BRC1* full sequences without codon stop were cloned in *pDONR207* and then inserted by Gateway cloning (Thermofisher) in *pABindGFP*, *pABindmCherry* and *pABindFRET* allowing production of the proteins fused to GFP, mCherry or GFP-mCherry, respectively. Vectors were Agro-infiltrated in *N*. *benthamiana* leaves and protein production was inducted 24h after infiltration and APB-FRET assays were performed 16-20h after induction. APB-FRET conditions and FRET efficiency were as described in Nicolas et al. [10].

### Transactivation assay

To perform the transactivation assay, we used a 2-kb promoter of the *BRC1* direct target gene *HB53* cloned in *pGWB435* [53] for fusion with the *LUCIFERASE* reporter gene as described in González-Grandío et al. [18]. *TIE1* and *BRC1* were cloned in the destination vector *pGWB2* for their constitutive expression under the *CaMV 35S* promoter. The different constructs were co-infiltrated in tobacco leaves and the LUC activity was measured 16–20 h after infiltration in a LB 960 Microplate Luminometer (Berthold) as described in Nicolas et al. [9].

### Accession numbers

Sequence data from this article can be found in the Arabidopsis Genome Initiative under the following accession numbers: TIE1, At4g28840; TIE2, AT2g20080; TIE3, At1g29010; TIE4, At2g34010; BRC1, AT3g18550; HB21, At2g18550; HB40, AT4g36740; HB53, AT5g66700.

## Supporting information

S1 Fig The shoot branching phenotype of *tie1-D*. (A) Branching phenotypes of 35-day-old wild-type plants and *tie1-D* mutants. Scale bar = 1 cm. (B) and (C) Close-up views of rosette leaf branches in the wild type and *tie1-D* mutant. Scale bars = 1 mm. (D) Number of primary rosette branches of 35-day-old wild-type plants and *tie1-D* mutants (n = 10).
(TIF)

S2 Fig TIE2 and TIE4 also interact with BRC1. Yeast two-hybrid assays of TIE2, TIE3 or TIE4 with BRC1. AD, activation domain; DBD, DNA binding domain. Co-transformed yeast cells were grown on medium lacking Leu and Trp (SD-Leu-Trp) or in selective medium lacking Leu, Trp and His (SD-Leu-Trp-His) with 2.5 mM 3-amino-1,2,4 triazole.
(TIF)

S3 Fig TIE1 may regulate *BRC1* at the transcriptional level. (A) The Fragments Per Kilobase Million (FPKM) Value of *BRC1* in RNA-seq. (B) The *BRC1* mRNA levels were quantified by qRT-PCR in wild type and *35S-GFP-TIE1*. (C) The *BRC1* mRNA levels were quantified by qRT-PCR in wild type and *35S-TIE1mEAR-VP16* lines. The expression was normalized to *AtUBQ10* levels, and was relative to wild-type levels. Data represents mean ± SD from three biological replicates. Significant differences are indicated ***p< 0.001 (two-tailed Student’s t-test).
(TIF)

S1 Table List of genes regulated by *TIE1*. (XLS)

S2 Table List of *BRC1*-dependent and *TIE1*-dependent genes. (XLS)

S3 Table List of bud activation and bud dormancy genes co-regulated by *TIE1* and *BRC1*. (XLS)

S4 Table List of primers used in this study. (XLS)
