# Correction: The TIE1 transcriptional repressor controls shoot branching by directly repressing BRANCHED1 in Arabidopsis

**DOI:** 10.1371/journal.pgen.1007565

**Published:** 2018-08-24

**Authors:** Yan Yang, Michael Nicolas, Jinzhe Zhang, Hao Yu, Dongshu Guo, Rongrong Yuan, Tiantian Zhang, Jianzhao Yang, Pilar Cubas, Genji Qin

There is an error in S4 Table. The forward and reverse primers for the *HB40* gene are duplicated. The authors have provided the correct version of S4 Table, which can be viewed below.

## Supporting information

S4 Table List of primers used in this study. (XLSX)
