# Supplementary figures and images for: The TIE1 transcriptional repressor controls shoot branching by directly repressing BRANCHED1 in Arabidopsis
Source: PLoS Genet. 2018 Mar 23;14(3):e1007296. doi: 10.1371/journal.pgen.1007296 (PMC5884558; doi:10.1371/journal.pgen.1007296)

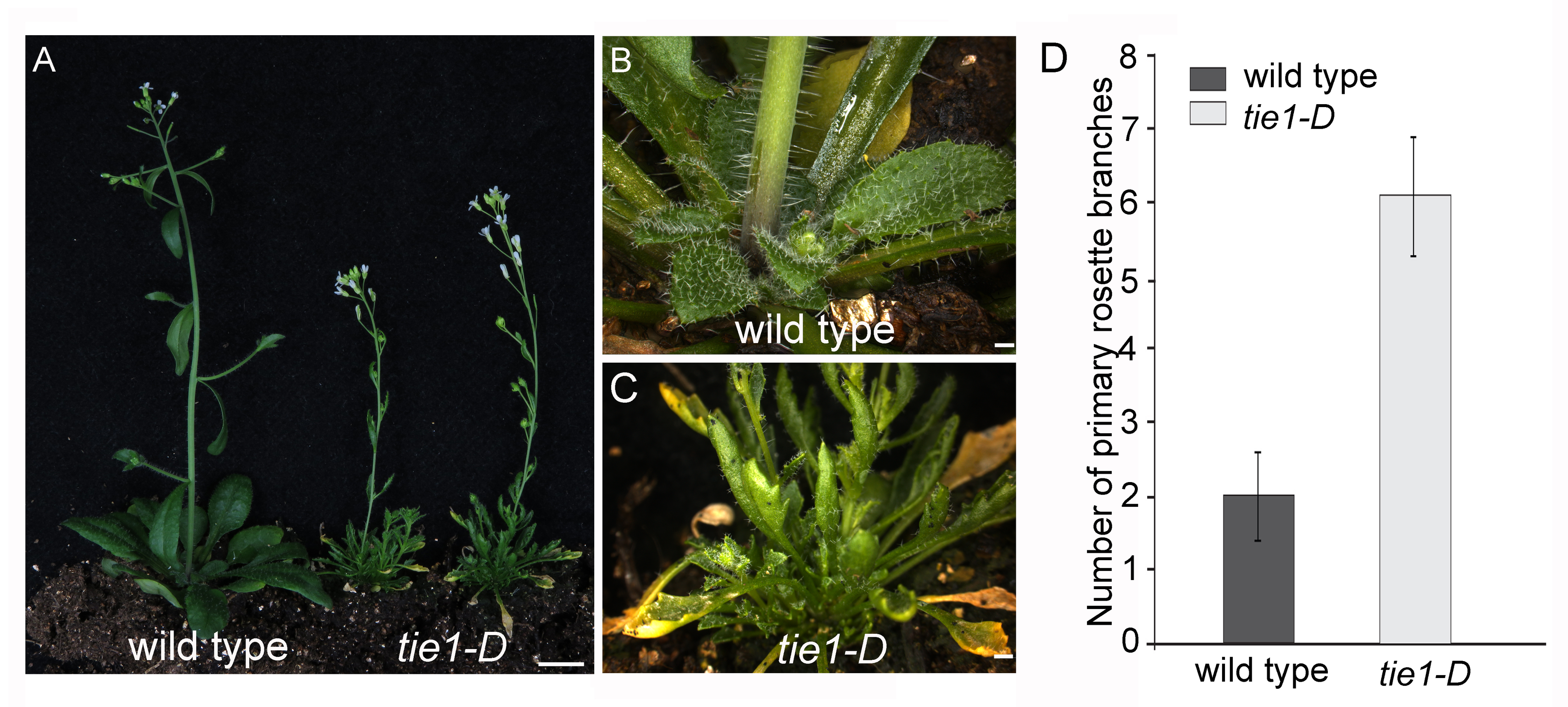

Supplement: S1 Fig — (A) Branching phenotypes of 35-day-old wild-type plants and tie1-D mutants. Scale bar = 1 cm. (B) and (C) Close-up views of rosette leaf branches in the wild type and tie1-D mutant. Scale bars = 1 mm. (D) Number of primary rosette branches of 35-day-old wild-type plants and tie1-D mutants (n = 10). (TIF) [file pgen.1007296.s001.tif]

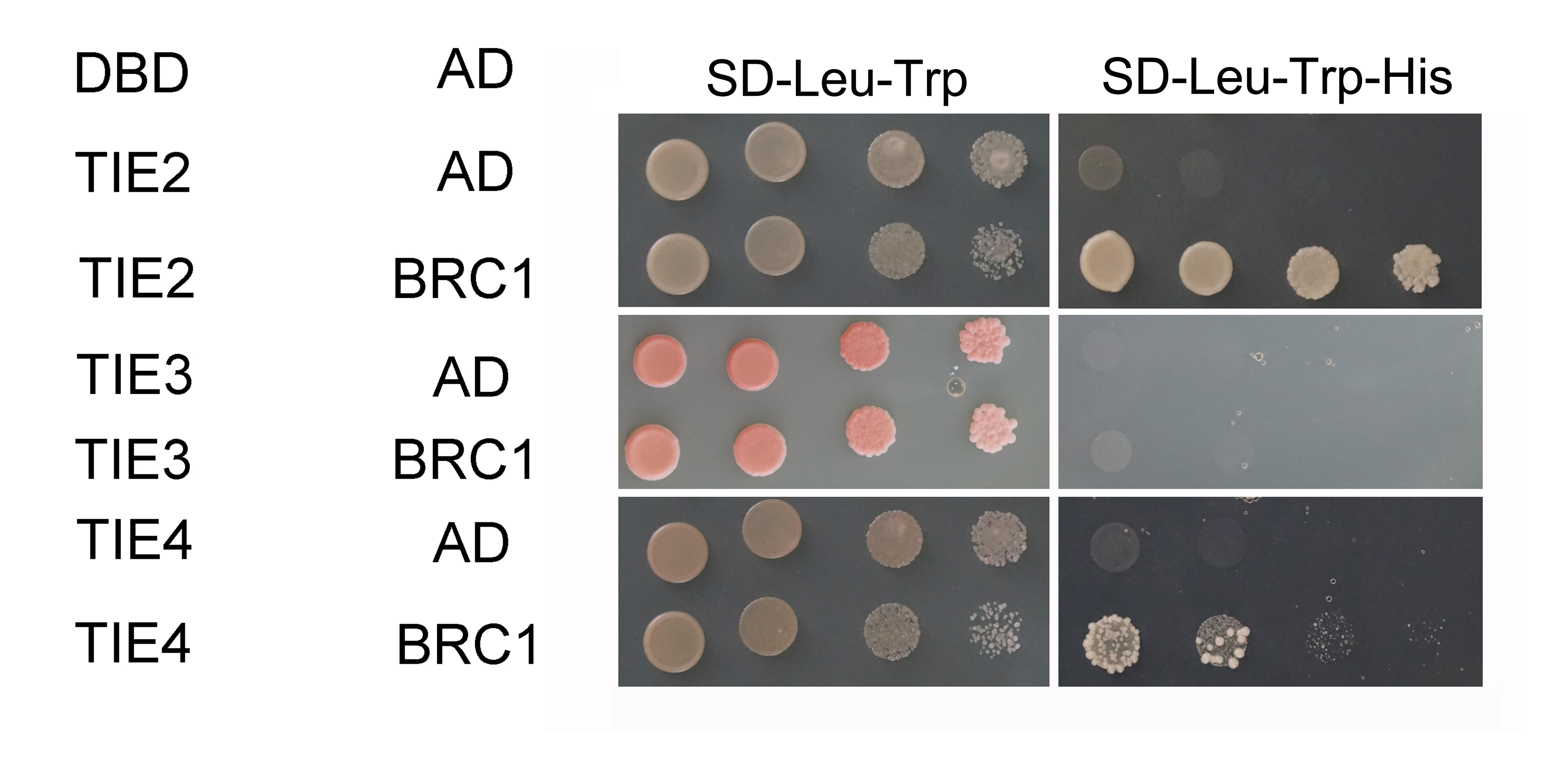

Supplement: S2 Fig — Yeast two-hybrid assays of TIE2, TIE3 or TIE4 with BRC1. AD, activation domain; DBD, DNA binding domain. Co-transformed yeast cells were grown on medium lacking Leu and Trp (SD-Leu-Trp) or in selective medium lacking Leu, Trp and His (SD-Leu-Trp-His) with 2.5 mM 3-amino-1,2,4 triazole. (TIF) [file pgen.1007296.s002.tif]

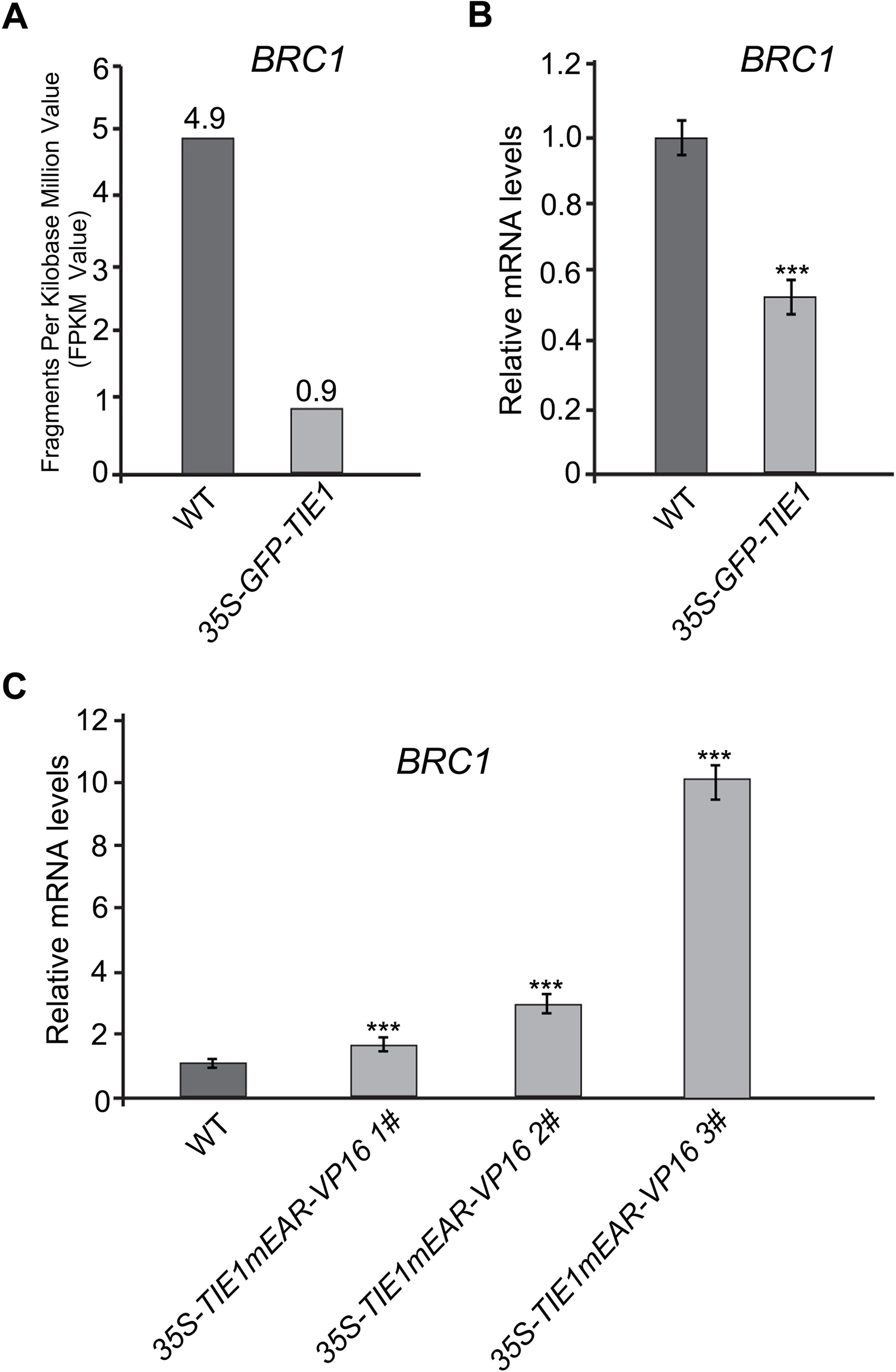

Supplement: S3 Fig — (A) The Fragments Per Kilobase Million (FPKM) Value of BRC1 in RNA-seq. (B) The BRC1 mRNA levels were quantified by qRT-PCR in wild type and 35S-GFP-TIE1. (C) The BRC1 mRNA levels were quantified by qRT-PCR in wild type and 35S-TIE1mEAR-VP16 lines. The expression was normalized to AtUBQ10 levels, and was relative to wild-type levels. Data represents mean ± SD from three biological replicates. Significant differences are indicated ***p< 0.001 (two-tailed Student’s t-test). (TIF) [file pgen.1007296.s003.tif]
